# Relationship Between QT Interval Parameters on Electrocardiography and Acute Cerebrovascular Accident: A Cross-Sectional Study

**DOI:** 10.7759/cureus.114358

**Published:** 2026-08-11

**Authors:** Paarth Sethi, Jagmeet Singh, Ravi Garg, Harsmeet Kaur Bhinder, Rohit Raina, Prabh Simranpal, Nandita Prabhat Tiwari, RR Singh, Dharmendra Uraiya

**Affiliations:** 1 Cardiology, Amrita Institute of Medical Sciences, Kochi, IND; 2 General Medicine, All India Institute of Medical Sciences, Bathinda, Bathinda, IND; 3 Nephrology, National Institute of Medical Sciences, Jaipur, IND; 4 Critical Care Medicine, Amrita Institute of Medical Sciences, Kochi, IND; 5 Emergency Medicine, Civil Hospital, Malout, IND; 6 Neurology, Hind Institute of Medical Sciences, Lucknow, IND; 7 General Medicine, Hind Institute of Medical Sciences, Lucknow, IND

**Keywords:** acute cerebrovascular accident, corrected qt interval, electrocardiography, modified rankin scale, qt dispersion

## Abstract

Introduction

Acute cerebrovascular accident (CVA) is a major cause of mortality and long-term disability worldwide. Electrocardiographic (ECG) abnormalities, particularly prolonged corrected QT interval (QTc) and QT dispersion (QTd), are frequently observed following acute stroke because of autonomic nervous system dysfunction. However, their clinical relevance in relation to functional outcome remains incompletely understood. This study aimed to compare QT interval parameters between ischemic and hemorrhagic stroke and determine the association of QTc and QTd with functional outcome.

Materials and methods

A hospital-based cross-sectional analytical study was conducted in the Department of General Medicine, Hind Institute of Medical Sciences, Barabanki, Uttar Pradesh, India, from January 2022 to December 2024. A total of 165 consecutive patients with acute CVA were enrolled, including 120 (72.7%) patients with ischemic stroke and 45 (27.3%) with hemorrhagic stroke. Standard 12-lead ECG was performed at admission to measure QT interval, QTc, QTmax, QTmin, QTd, and corrected QTd. Functional outcome was assessed using the modified Rankin Scale (mRS). Statistical analysis was performed using IBM SPSS Statistics for Windows, Version 26.0 (Released 2018; IBM Corp., Armonk, NY, USA), with a p*-*value <0.05 considered statistically significant.

Results

The mean QT interval parameters were comparable between ischemic and hemorrhagic stroke (all p > 0.05). Prolonged QTc (>0.44 s) was more common in hemorrhagic stroke, occurring in 21 (46.7%) patients, whereas QTd >0.08 s was observed in 69 (57.5%) patients with ischemic stroke and 26 (57.8%) patients with hemorrhagic stroke. Severe disability (mRS 4-5) occurred in 53 (44.2%) patients with ischemic stroke and 18 (40.0%) patients with hemorrhagic stroke. Significant associations were observed between QTc category and functional outcome (p < 0.001), as well as between QTd and functional outcome (p < 0.001) in both stroke subtypes. QTc (r = 0.435, p < 0.001) and QTd (r = 0.317, p < 0.001) demonstrated significant positive correlations with mRS scores.

Conclusion

QT interval abnormalities are common in patients with acute CVA. Prolonged QTc interval and increased QTd were significantly associated with poorer functional outcomes. These findings suggest that QTc and QTd may serve as simple, readily available, and non-invasive markers associated with functional outcomes in patients with acute stroke. However, prospective longitudinal studies with multivariable adjustment are required to establish their independent prognostic value.

## Introduction

Stroke, also known as cerebrovascular accident (CVA), is defined as the sudden onset of neurological dysfunction resulting from cerebral ischemia or hemorrhage and remains a major cause of mortality and long-term disability worldwide [1,2]. Ischemic stroke accounts for nearly 80%-85% of all stroke cases, while hemorrhagic stroke comprises the remaining proportion but is associated with higher morbidity and mortality [3,4]. Despite advances in diagnostic imaging and acute stroke management, stroke continues to impose a substantial clinical and socioeconomic burden, particularly in low- and middle-income countries, including India, where the incidence and prevalence have increased with demographic and epidemiological transition [5].

Acute stroke is frequently associated with cardiovascular abnormalities resulting from disturbances in autonomic nervous system regulation [6]. Cerebral injury, particularly involving autonomic regulatory centers, may lead to excessive sympathetic activation and reduced parasympathetic activity, producing a variety of electrocardiographic (ECG) abnormalities [7]. These include QT interval prolongation, QT dispersion (QTd), ST-segment changes, T-wave abnormalities, and cardiac arrhythmias [8]. Such ECG changes are observed in a substantial proportion of patients with both ischemic and hemorrhagic stroke and may occur even in the absence of underlying structural heart disease [9].

Among these ECG abnormalities, corrected QT interval (QTc) and QTd have attracted considerable attention because they reflect ventricular repolarization abnormalities and autonomic dysfunction [10]. QTd, defined as the difference between the maximum and minimum QT intervals on a standard 12-lead ECG, is considered an indirect marker of ventricular repolarization heterogeneity [8]. Several studies have demonstrated an association between prolonged QTc or increased QTd and greater stroke severity, poor functional recovery, cardiac complications, and increased mortality [11-13]. However, published evidence remains inconsistent owing to differences in study populations, limited sample sizes, heterogeneous stroke subtypes, and variations in ECG measurement techniques.

Given the potential clinical relevance of ECG parameters and the limited evidence from the Indian population, further evaluation of QT interval abnormalities in patients with acute CVA is warranted. Therefore, the present study aimed to compare QT interval parameters between ischemic and hemorrhagic stroke and to determine the association of QTc and QTd with functional outcome, as assessed by the modified Rankin Scale (mRS).

## Materials and methods

This hospital-based cross-sectional analytical study was conducted in the Department of General Medicine, Hind Institute of Medical Sciences, Safedabad, Barabanki, Uttar Pradesh, India, from January 2022 to December 2024, after obtaining approval from the Institutional Ethics Committee (IEC No. HIMS/IRB/2021-22/3752). Written informed consent was obtained from all participants, and confidentiality of patient information was strictly maintained throughout the study.

The sample size was estimated using the single population proportion formula, based on the reported 29% prevalence of prolonged QTc interval by Sebastian and Roy [13]. The formula used was: $n=\frac{Z_{\alpha}^{2}p(1-p)}{L^{2}}$, where n is the required sample size, Zα is the standard normal deviate corresponding to the desired confidence level (1.96 for 95% confidence), p is the expected prevalence (0.29), and L is the allowable absolute error (0.07). The calculated minimum sample size was rounded up, and 165 patients were enrolled in the study.

A total of 165 consecutive patients presenting with acute CVA were included in the study. Patients with metabolic causes of sudden loss of consciousness, brain tumors, head trauma, or those receiving medications known to prolong the QT interval (including antiarrhythmic, antimalarial, and psychotropic drugs) were excluded. Consecutive sampling was employed to recruit eligible patients admitted to the emergency and inpatient General Medicine services during the study period. Baseline demographic variables, including age, sex, occupation, comorbidities, presenting complaints, duration from symptom onset to hospital presentation, and site of lesion, were recorded using a structured case record form. Stroke severity using the National Institutes of Health Stroke Scale (NIHSS) was not routinely documented during the study period and was therefore not included in the analysis.

All patients underwent detailed clinical evaluation. The diagnosis and classification of stroke into ischemic or hemorrhagic stroke were confirmed using computed tomography (CT) of the brain (16-slice GE scanner; GE Healthcare, Milwaukee, WI, USA) and/or magnetic resonance imaging (MRI) (1.5-T Philips Ingenia scanner; Philips Healthcare, Best, The Netherlands) at presentation. A standard 12-lead electrocardiogram (ECG) was obtained at admission using a TRICOG MAC 600 ECG machine (GE Healthcare, Milwaukee, WI, USA) at a paper speed of 25 mm/s and calibration of 10 mm/mV. QT interval was manually measured by a single trained investigator from the onset of the QRS complex to the end of the T wave in three to five consecutive complexes in lead II, and the average value was used for analysis. QT interval parameters evaluated included QT interval, QTc interval using Bazett's formula $QTc = \frac{QT}{\sqrt{RR}}$, QTmax, QTmin, QTd (QTmax - QTmin), and QTc dispersion (QTcd). QTc was categorized into <0.40 s, 0.40-0.44 s, and >0.44 s, while QTd was categorized into <0.05 s, 0.05-0.08 s, and >0.08 s for subgroup analysis [14].

Functional outcome was assessed at the time of hospital discharge using the mRS and classified as slight disability (mRS 1-2), moderate disability (mRS 3), severe disability (mRS 4-5), and death (mRS 6) [15]. The distribution of QT interval abnormalities was compared between ischemic and hemorrhagic stroke, and the association of QTc categories and QTd categories with functional outcome was evaluated separately in both stroke groups. In addition, the correlation between QT interval parameters and mRS scores was determined using Spearman rank correlation analysis.

Data were entered into Microsoft Excel (Microsoft® Corp., Redmond, WA, USA) and analyzed using IBM SPSS Statistics for Windows, Version 26.0 (Released 2018; IBM Corp., Armonk, NY, USA). Continuous variables were expressed as mean ± standard deviation (SD), whereas categorical variables were summarized as frequency and percentage (N (%)). Comparisons of continuous variables between ischemic and hemorrhagic stroke were performed using the independent Student's t-test, whereas categorical variables were analyzed using the Chi-square test. The relationship between QT interval parameters and functional outcome was assessed using Spearman's rank correlation coefficient. No multivariable regression analysis was performed because important potential confounding variables, including NIHSS, electrolyte measurements, and echocardiographic findings, were not uniformly available for all participants. A p-value <0.05 was considered statistically significant.

## Results

A total of 165 patients with acute CVA were included in the study, comprising 120 (72.7%) patients with ischemic stroke and 45 (27.3%) patients with hemorrhagic stroke. The mean age was comparable between patients with ischemic and hemorrhagic stroke (57.28 ± 16.56 vs. 57.13 ± 11.59 years; p=0.956). Males constituted 63 (52.5%) patients with ischemic stroke, while females accounted for 24 (53.3%) patients with hemorrhagic stroke. Housewives were the predominant occupational group in both cohorts, and hypertension was the most common comorbidity, observed in 42 (35.0%) patients with ischemic stroke and 28 (62.2%) patients with hemorrhagic stroke. Occupation (p=0.001) and comorbidity profile (p=0.033) differed significantly between the groups (Table 1).

**Table 1:** Baseline demographic characteristics of patients with acute cerebrovascular accident Data are presented as n (%) for categorical variables and mean ± standard deviation (SD) for continuous variables. Comparisons between ischemic and hemorrhagic stroke groups were performed using the Chi-square (χ²) test for categorical variables and the independent Student's t-test for continuous variables. A p-value <0.05 was considered statistically significant. SD, standard deviation; χ², Chi-square statistic; t, Student's t statistic; CKD, chronic kidney disease; CVA, cerebrovascular accident

Variable	Category	Ischemic stroke (n = 120)	Hemorrhagic stroke (n = 45)	Test statistic	p-value
Age group (years)	17-29	5 (4.2%)	0 (0.0%)	χ²=5.91	0.315
	30-39	7 (5.8%)	3 (6.7%)		
	40-49	18 (15.0%)	6 (13.3%)		
	50-59	27 (22.5%)	15 (33.3%)		
	60-69	24 (20.0%)	12 (26.7%)		
	70-80	39 (32.5%)	9 (20.0%)		
	Mean ± SD	57.28 ± 16.56	57.13 ± 11.59	t=0.056	0.956
Gender	Male	63 (52.5%)	21 (46.7%)	χ²=0.446	0.504
	Female	57 (47.5%)	24 (53.3%)		
Occupation	Farmer	21 (17.5%)	6 (13.3%)	χ²=19.42	0.001
	Housewife	51 (42.5%)	21 (46.7%)		
	Labor	12 (10.0%)	15 (33.3%)		
	Service	12 (10.0%)	0 (0.0%)		
	Shopkeeper	24 (20.0%)	3 (6.7%)		
Comorbidities	Hypertension	42 (35.0%)	28 (62.2%)	χ²=25.15	0.033
	Diabetes mellitus	36 (30.0%)	5 (11.1%)		
	Smoking	12 (10.0%)	3 (6.7%)		
	Previous CVA	7 (5.8%)	0 (0.0%)		
	CKD	6 (5.0%)	2 (4.4%)		
	No comorbidity	21 (17.5%)	8 (17.8%)		

Right-sided weakness was the most common presenting complaint in both ischemic stroke (57 (47.5%)) and hemorrhagic stroke (21 (46.7%)). The left hemisphere was the commonest site of lesion in both groups. Patients with ischemic stroke had a significantly longer duration of illness than those with hemorrhagic stroke (1.28 ± 0.57 vs. 1.04 ± 0.51 days; p = 0.014) (Table 2).

**Table 2:** Clinical characteristics of study participants Data are presented as n (%) for categorical variables and mean ± standard deviation (SD) for duration of illness. Comparisons between ischemic and hemorrhagic stroke groups were performed using the Chi-square (χ²) test for categorical variables and the independent Student's t-test for continuous variables. *A p-value <0.05 was considered statistically significant. SD, standard deviation; χ², Chi-square statistic; t, Student's t statistic

Variable	Category	Ischemic stroke (n=120)	Hemorrhagic stroke (n=45)	Test statistic	p-value
Presenting complaint	Right-sided weakness	57 (47.5%)	21 (46.7%)	χ²=7.08	0.132
	Left-sided weakness	39 (32.5%)	15 (33.3%)		
	Aphasia	3 (2.5%)	0 (0.0%)		
	Loss of consciousness	12 (10.0%)	9 (20.0%)		
	Seizures	9 (7.5%)	0 (0.0%)		
Site of lesion	Left	46 (38.3%)	24 (53.3%)	χ²=7.37	0.118
	Right	43 (35.8%)	16 (35.6%)		
	Posterior	11 (9.2%)	4 (8.9%)		
	Anterior	9 (7.5%)	1 (2.2%)		
	Bilateral lacunar infarct	11 (9.2%)	0 (0.0%)		
Duration of illness (days)	0	7 (5.8%)	5 (11.1%)	χ²=6.06	0.048
	1	72 (60.0%)	33 (73.3%)		
	2	41 (34.2%)	7 (15.6%)		
	Mean ± SD	1.28 ± 0.57	1.04 ± 0.51	t=2.48	0.014*

The mean QT interval parameters, including QT, QTc, QTmax, QTmin, QTd, and QTcd, were comparable between ischemic and hemorrhagic stroke, with no statistically significant differences (all p>0.05) (Table 3).

**Table 3:** Comparison of QT interval parameters between ischemic and hemorrhagic stroke Data are presented as mean ± standard deviation (SD). Comparisons between ischemic and hemorrhagic stroke groups were performed using the independent Student's t-test. A p-value <0.05 was considered statistically significant. SD, standard deviation; QTc, corrected QT interval; QTmax, maximum QT interval; QTmin, minimum QT interval; QTd, QT dispersion; QTcd, corrected QT dispersion; t, Student's t statistic

Parameter	Ischemic stroke (n=120)	Hemorrhagic stroke (n=45)	Test statistic	p-value
QT interval (s)	0.37 ± 0.06	0.38 ± 0.06	t=0.95	0.342
QTc (s)	0.44 ± 0.05	0.44 ± 0.05	t=0.00	>0.999
QTmax (s)	0.40 ± 0.06	0.41 ± 0.07	t=0.91	0.364
QTmin (s)	0.33 ± 0.05	0.34 ± 0.05	t=1.14	0.254
QTd (s)	0.07 ± 0.03	0.07 ± 0.03	t=0.00	>0.999
QTcd (s)	0.11 ± 0.15	0.12 ± 0.17	t=0.37	0.714

Among patients with ischemic stroke, QTc values of 0.40-0.44 s were most frequent (53 (44.2%)), whereas QTc >0.44 s predominated in hemorrhagic stroke (21 (46.7%)). Increased QTd (>0.08 s) was observed in 69 (57.5%) patients with ischemic stroke and 26 (57.8%) patients with hemorrhagic stroke. However, the distribution of QT interval abnormalities did not differ significantly between the two groups (all p>0.05) (Table 4).

**Table 4:** Distribution of QT interval abnormalities Data are presented as n (%). Comparisons between ischemic and hemorrhagic stroke groups were performed using the Chi-square (χ²) test. A p-value <0.05 was considered statistically significant. QTc, corrected QT interval; QTmax, maximum QT interval; QTmin, minimum QT interval; χ², Chi-square statistic

Variable	Category	Ischemic stroke (n=120)	Hemorrhagic stroke (n=45)	χ²	p-value
QTc	<0.40 s	24 (20.0%)	10 (22.2%)	2.44	0.295
	0.40-0.44 s	53 (44.2%)	14 (31.1%)		
	>0.44 s	43 (35.8%)	21 (46.7%)		
QTmax	<0.40 s	41 (34.2%)	18 (40.0%)	5.58	0.062
	0.40-0.44 s	62 (51.7%)	15 (33.3%)		
	>0.44 s	17 (14.2%)	12 (26.7%)		
QTmin	<0.40 s	101 (84.2%)	35 (77.8%)	0.92	0.337
	0.40-0.44 s	19 (15.8%)	10 (22.2%)		
QTd	<0.05 s	45 (37.5%)	17 (37.8%)	0.02	0.989
	0.05-0.08 s	6 (5.0%)	2 (4.4%)		
	>0.08 s	69 (57.5%)	26 (57.8%)		

Severe disability (mRS 4-5) was the most common functional outcome in both ischemic stroke (53 (44.2%)) and hemorrhagic stroke (18 (40.0%)). Functional outcomes were comparable between the two groups (p=0.567) (Table 5).

**Table 5:** Functional outcome according to modified Rankin Scale Data are presented as n (%). Comparisons of functional outcomes between ischemic and hemorrhagic stroke groups were performed using the Chi-square (χ²) test. A p-value <0.05 was considered statistically significant. mRS, modified Rankin Scale; χ², chi-square statistic

Outcome	Ischemic stroke (n=120)	Hemorrhagic stroke (n=45)	χ²	p-value
Slight disability (mRS 1-2)	39 (32.5%)	13 (28.9%)	2.03	0.567
Moderate disability (mRS 3)	25 (20.8%)	11 (24.4%)		
Severe disability (mRS 4-5)	53 (44.2%)	18 (40.0%)		
Death (mRS 6)	3 (2.5%)	3 (6.7%)		

Among patients with ischemic stroke, severe disability was most common in those with QTc of 0.40-0.44 s (28 (52.8%)), while increased QTd (>0.08 s) was associated with severe disability in 31 (44.9%) patients and all deaths (3 (100.0%)). Both QTc category and QTd category showed significant associations with functional outcome (both p<0.001) (Table 6).

**Table 6:** Association between QTc interval and QTd with functional outcome among patients with ischemic stroke (n = 120) Data are presented as n (%). Associations between QTc interval categories, QT dispersion categories, and functional outcomes among patients with ischemic stroke were analyzed using the Chi-square (χ²) test. A p-value <0.05 was considered statistically significant. QTc, corrected QT interval; mRS, modified Rankin Scale; χ², Chi-square statistic

Variable	Category	Slight disability (mRS 1-2) n=39	Moderate disability (mRS 3) n=25	Severe disability (mRS 4-5) n=53	Death (mRS 6) n=3	χ²	p-value
QTc category	<0.40 s (n=24)	17 (70.8%)	2 (8.3%)	5 (20.8%)	0 (0.0%)	29.17	< 0.001
	0.40-0.44 s (n=53)	16 (30.2%)	7 (13.2%)	28 (52.8%)	2 (3.8%)		
	>0.44 s (n=43)	6 (14.0%)	16 (37.2%)	20 (46.5%)	1 (2.3%)		
QTd	<0.05 s (n=45)	22 (48.9%)	1 (2.2%)	22 (48.9%)	0 (0.0%)	27.02	< 0.001
	0.05-0.08 s (n=6)	2 (33.3%)	4 (66.7%)	0 (0.0%)	0 (0.0%)		
	>0.08 s (n=69)	15 (21.7%)	20 (29.0%)	31 (44.9%)	3 (4.3%)		

Among patients with hemorrhagic stroke, QTc >0.44 s was associated with severe disability in 13 (61.9%) patients. Similarly, increased QTd (>0.08 s) was associated with severe disability in 13 (50.0%) patients and all deaths (3 (11.5%)). Both QTc category and QTd category were significantly associated with functional outcome (both p<0.001) (Table 7).

**Table 7:** Association between QTc interval and QTd with functional outcome among patients with hemorrhagic stroke (n = 45) Data are presented as n (%). Associations between QTc interval categories, QT dispersion categories, and functional outcomes among patients with hemorrhagic stroke were analyzed using the Chi-square (χ²) test. A p-value <0.05 was considered statistically significant. QTc, corrected QT interval; mRS, modified Rankin Scale; χ², Chi-square statistic

Variable	Category	Slight disability (mRS 1-2) n=13	Moderate disability (mRS 3) n=11	Severe disability (mRS 4-5) n=18	Death (mRS 6) n=3	χ²	p-value
QTc category	<0.40 s (n=10)	3 (30.0%)	6 (60.0%)	1 (10.0%)	0 (0.0%)	16.52	< 0.001
	0.40-0.44 s (n=14)	7 (50.0%)	2 (14.3%)	4 (28.6%)	1 (7.1%)		
	>0.44 s (n=21)	3 (14.3%)	3 (14.3%)	13 (61.9%)	2 (9.5%)		
QTd	<0.05 s (n=17)	8 (47.1%)	4 (23.5%)	5 (29.4%)	0 (0.0%)	12.44	< 0.001
	0.05-0.08 s (n=2)	0 (0.0%)	2 (100.0%)	0 (0.0%)	0 (0.0%)		
	>0.08 s (n=26)	5 (19.2%)	5 (19.2%)	13 (50.0%)	3 (11.5%)		

Spearman correlation analysis demonstrated significant positive correlations between functional outcome and QTc (r=0.435, p<0.001), as well as QTd (r=0.317, p<0.001), demonstrating a significant association between greater QT interval abnormalities and higher mRS scores (Table 8).

**Table 8:** Correlation between QT interval parameters and functional outcome Data are presented as Spearman's correlation coefficient (r) with corresponding 95% confidence intervals (CI). Correlations between QT interval parameters and functional outcome were assessed using Spearman's rank correlation analysis. A p-value <0.05 was considered statistically significant. QTc, corrected QT interval; mRS, modified Rankin Scale; CI, confidence interval; r, Spearman's correlation coefficient

Variable	Spearman's correlation (r)	95% confidence interval	p-value
Outcome (mRS) vs QTc	0.435	0.299-0.555	<0.001
Outcome (mRS) vs QTd	0.317	0.169-0.452	<0.001

## Discussion

The present study evaluated the relationship between QT interval parameters and functional outcomes in patients with acute CVA. The mean age was comparable between ischemic and hemorrhagic stroke patients (57.28 ± 16.56 vs. 57.13 ± 11.59 years). Male predominance was observed in ischemic stroke patients, 63 (52.5%), whereas females constituted the majority in hemorrhagic stroke patients, 24 (53.3%). Hypertension was the most frequent comorbidity in both ischemic stroke patients, 42 (35.0%), and hemorrhagic stroke patients, 28 (62.2%), followed by diabetes mellitus (ischemic: 36 (30.0%); hemorrhagic: 5 (11.1%)). These findings are consistent with previous studies that reported increasing stroke incidence with advancing age, a predominance of male patients, and hypertension as the most important vascular risk factor for both ischemic and hemorrhagic stroke [16-18]. Similar observations have also been reported in the INTERSTROKE study, emphasizing hypertension as the strongest modifiable risk factor for stroke worldwide [19].

Right-sided weakness was the most common presenting complaint in both ischemic (57 (47.5%)) and hemorrhagic stroke (21 (46.7%)), followed by left-sided weakness (ischemic: 33 (27.5%); hemorrhagic: 15 (33.3%)). Left hemispheric involvement was the predominant site of lesion in both ischemic (43 (35.8%)) and hemorrhagic stroke (27 (60.0%)) patients. Most patients presented within one day of symptom onset (ischemic: 72 (60.0%); hemorrhagic: 33 (73.3%)), reflecting early hospital admission. These observations are comparable with previous studies, which also identified hemiparesis as the predominant clinical manifestation of acute stroke and reported left hemispheric lesions more frequently than right-sided lesions [18,20]. Similar studies have likewise demonstrated hypertension and diabetes mellitus as the leading comorbid conditions contributing to stroke occurrence [21,22].

The principal finding of the present study was the high prevalence of QT interval abnormalities among patients with acute CVA. The mean QT interval was 0.37 ± 0.06 s in ischemic stroke and 0.38 ± 0.06 s in hemorrhagic stroke, while the mean QTc was 0.44 ± 0.05 s in both groups. QTd was also comparable (0.07 ± 0.03 s), although QTmax and QTcd were slightly higher among hemorrhagic stroke patients. Most ischemic stroke patients had QTc values between 0.40 and 0.44 s (53 (44.2%)), whereas prolonged QTc (>0.44 s) predominated among hemorrhagic stroke patients 21 (46.7%). Increased QTd (>0.08 s) was observed in ischemic 69 (57.5%) and hemorrhagic 26 (57.8%) patients. These findings support previous reports demonstrating that ventricular repolarization abnormalities are common following acute stroke and are likely mediated through autonomic dysfunction [16,23]. Previous investigators have similarly reported higher QTc and QTd values among patients with acute ischemic stroke compared with healthy controls, suggesting that QT interval abnormalities reflect altered cardiac autonomic regulation after cerebrovascular injury [16,24].

Assessment of functional outcome revealed that major disability (mRS 4-5) was the most frequent outcome in both ischemic 53 (44.2%) and hemorrhagic 18 (40.0%) stroke patients, whereas mortality occurred in 3 (2.5%) ischemic and 3 (6.7%) hemorrhagic stroke patients. Patients with prolonged QTc and increased QTd showed a higher frequency of severe disability. Among ischemic stroke patients with major disability, 28 (52.8%) had QTc values between 0.40 and 0.44 s, whereas 13 (72.2%) hemorrhagic stroke patients with major disability had QTc values >0.44 s. Similarly, QTd >0.08 s was observed in 31 (58.5%) ischemic and 13 (72.2%) hemorrhagic stroke patients with major disability. Furthermore, both QTc (r = 0.435; p < 0.001) and QTd (r = 0.317; p < 0.001) demonstrated significant positive correlations with mRS scores, indicating a significant association between greater ventricular repolarization abnormalities and worse functional status at hospital discharge. These findings are in agreement with previous studies that demonstrated significant associations between prolonged QTc, increased QTd, stroke severity, and adverse neurological outcomes [24-26]. A study reported that persistent QT prolongation after acute ischemic stroke was associated with increased mortality, while other investigators have shown QTd to be a useful marker associated with functional outcome following acute neurological injury [25,26]. However, because the present study employed a cross-sectional design, these findings should be interpreted as associations rather than evidence of an independent prognostic or causal relationship. Furthermore, a previous study failed to demonstrate an independent prognostic significance of QT prolongation after adjustment for stroke severity and associated comorbidities, suggesting that infarct size, lesion location, and underlying cardiovascular disease may also influence patient outcomes [20].

Strengths and limitations

The present study has several strengths. It included a relatively adequate sample of patients with both ischemic and hemorrhagic stroke, allowing comparison between the two major stroke subtypes. QT interval parameters were measured using a standardized 12-lead electrocardiogram in all participants, and functional outcomes were assessed using the validated mRS, enabling objective evaluation of the relationship between ECG abnormalities and neurological outcome. The study also comprehensively evaluated multiple QT interval parameters, including QT, QTc, QTmax, QTmin, QTd, and QTcd, thereby providing a broader assessment of ventricular repolarization abnormalities in acute CVA.

However, certain limitations should be acknowledged. Being a single-center cross-sectional study, the findings may have limited generalizability to other populations. The relatively small number of patients with hemorrhagic stroke may also have limited statistical power for subgroup analyses. Serial ECG recordings and long-term follow-up were not performed; therefore, the temporal evolution of QT interval abnormalities and their association with long-term outcomes could not be assessed. Stroke severity was not assessed using the NIHSS, precluding adjustment for baseline neurological deficit. Manual ECG measurements were performed by a single observer, and intraobserver and interobserver variability were not assessed, introducing the possibility of observer bias. Serum electrolyte measurements were not uniformly available at the time of ECG acquisition and therefore could not be included in the analysis, although electrolyte abnormalities may influence QT interval measurements. In addition, autonomic function tests, cardiac biomarkers, and detailed echocardiographic evaluation were not routinely incorporated, making it difficult to completely exclude the influence of underlying subclinical cardiac disease on QT interval changes. Finally, multivariable regression analysis was not performed because important potential confounders, including NIHSS, electrolyte measurements, and echocardiographic findings, were unavailable for all participants. Consequently, the observed associations should be interpreted with appropriate caution.

## Conclusions

The present study demonstrated that ECG QT interval abnormalities are common in patients with acute CVA. Although mean QT interval parameters were comparable between ischemic and hemorrhagic stroke, prolonged QTc interval was more frequently observed in hemorrhagic stroke, while increased QTd was common in both stroke subtypes. Significant positive associations were observed between QTc, QTd, and functional outcome, suggesting that greater ventricular repolarization abnormalities are associated with worse functional status at hospital discharge. These findings suggest that QT interval parameters, particularly QTc and QTd, may serve as simple, readily available, and non-invasive adjunctive markers associated with functional outcome in patients with acute stroke. However, prospective longitudinal studies with multivariable adjustment are required to establish their independent prognostic value.

## References

[1] Kumar S, Sharma GD, Dogra VD (2017). A study of electrocardiogram changes in patients with acute stroke. Int J Res Med Sci.

[2] Murphy SJ, Werring DJ (2020). Stroke: causes and clinical features. Medicine (Abingdon).

[3] Thomas SE, Plumber N, Venkatapathappa P, Gorantla V (2021). A review of risk factors and predictors for hemorrhagic transformation in patients with acute ischemic stroke. Int J Vasc Med.

[4] Donkor ES (2018). Stroke in the 21st century: a snapshot of the burden, epidemiology, and quality of life. Stroke Res Treat.

[5] Kamalakannan S, Gudlavalleti AS, Gudlavalleti VS, Goenka S, Kuper H (2017). Incidence & prevalence of stroke in India: a systematic review. Indian J Med Res.

[6] Barkhudaryan A, Doehner W, Jauert N (2025). Autonomic dysfunction after stroke: an overview of recent clinical evidence and perspectives on therapeutic management. Clin Auton Res.

[7] Mo J, Huang L, Peng J, Ocak U, Zhang J, Zhang JH (2019). Autonomic disturbances in acute cerebrovascular disease. Neurosci Bull.

[8] Lederman YS, Balucani C, Lazar J, Steinberg L, Gugger J, Levine SR (2014). Relationship between QT interval dispersion in acute stroke and stroke prognosis: a systematic review. J Stroke Cerebrovasc Dis.

[9] Togha M, Sharifpour A, Ashraf H, Moghadam M, Sahraian MA (2013). Electrocardiographic abnormalities in acute cerebrovascular events in patients with/without cardiovascular disease. Ann Indian Acad Neurol.

[10] Alabd AA, Fouad A, Abdel-Nasser R, Nammas W (2009). QT interval dispersion pattern in patients with acute ischemic stroke: does the site of infarction matter?. Int J Angiol.

[11] Soliman EZ, Howard G, Cushman M (2012). Prolongation of QTc and risk of stroke: the REGARDS (REasons for Geographic and Racial Differences in Stroke) study. J Am Coll Cardiol.

[12] Wu J, Nizhamuding D, Liu P (2019). QT interval prolongation in patients with acute ischemic stroke: a report in northwest China. J Int Med Res.

[13] Sebastian S, Roy JS (2020). Electocardiographic changes inacute cerebrovascular accidents - a tertiary centre experience in South India. J Nutr Metab Health Sci.

[14] Malik M, Färbom P, Batchvarov V, Hnatkova K, Camm AJ (2002). Relation between QT and RR intervals is highly individual among healthy subjects: implications for heart rate correction of the QT interval. Heart.

[15] van Swieten JC, Koudstaal PJ, Visser MC, Schouten HJ, van Gijn J (1988). Interobserver agreement for the assessment of handicap in stroke patients. Stroke.

[16] Familoni OB, Odusan O, Ogun SA (2006). The pattern and prognostic features of QT intervals and dispersion in patients with acute ischemic stroke. J Natl Med Assoc.

[17] Tang H, Sun J, Wang Y (2020). QT interval dispersion as a predictor of clinical outcome in acute ischemic stroke. Front Neurol.

[18] Si Larbi MT, Al Mangour W, Saba I, Al Naqeb D, Faisal ZS, Omar S, Ibrahim F (2021). Ischemic and non-ischemic stroke in young adults - a look at risk factors and outcome in a developing country. Cureus.

[19] O'Donnell MJ, Chin SL, Rangarajan S (2016). Global and regional effects of potentially modifiable risk factors associated with acute stroke in 32 countries (INTERSTROKE): a case-control study. Lancet.

[20] Amin OS, Sheikhbzeni AS, Siddiq AN (2020). Relationship of QTc interval prolongation with acute ischemic stroke. Med Arch.

[21] Al Dawish MA, Robert AA, Braham R, Al Hayek AA, Al Saeed A, Ahmed RA, Al Sabaan FS (2016). Diabetes mellitus in Saudi Arabia: a review of the recent literature. Curr Diabetes Rev.

[22] Chen R, Ovbiagele B, Feng W (2016). Diabetes and stroke: epidemiology, pathophysiology, pharmaceuticals and outcomes. Am J Med Sci.

[23] Hanci V, Gül S, Dogan SM, Turan IO, Kalayci M, Açikgöz B (2010). Evaluation of P wave and corrected QT dispersion in subarachnoid haemorrhage. Anaesth Intensive Care.

[24] Lazar J, Busch D, Wirkowski E, Clark LT, Salciccioli L (2008). Changes in QT dispersion after thrombolysis for stroke. Int J Cardiol.

[25] Hromádka M, Seidlerová J, Rohan V (2016). Prolonged corrected QT interval as a predictor of clinical outcome in acute ischemic stroke. J Stroke Cerebrovasc Dis.

[26] Chao CC, Wang TL, Chong CF (2009). Prognostic value of QT parameters in patients with acute hemorrhagic stroke: a prospective evaluation with respect to mortality and post-hospitalization bed confinement. J Chin Med Assoc.

